# Massively-multiplexed epitope mapping techniques for viral antigen discovery

**DOI:** 10.3389/fimmu.2023.1192385

**Published:** 2023-09-25

**Authors:** Diya Hu, Aaron T. Irving

**Affiliations:** ^1^ Zhejiang University-University of Edinburgh Institute, Zhejiang University School of Medicine, Zhejiang University, Haining, China; ^2^ Department of Clinical Laboratory Studies, Second Affiliated Hospital, Zhejiang University School of Medicine, Hangzhou, China; ^3^ Centre for Infection, Immunity & Cancer, Zhejiang University-University of Edinburgh Institute, Zhejiang University School of Medicine, Zhejiang University, Haining, China; ^4^ Biomedical and Health Translational Research Centre of Zhejiang Province (BIMET), Haining, China; ^5^ College of Medicine & Veterinary Medicine, The University of Edinburgh, Edinburgh, United Kingdom

**Keywords:** epitope, epitope mapping, phage-display, T cell, B cell

## Abstract

Following viral infection, viral antigens bind specifically to receptors on the surface of lymphocytes thereby activating adaptive immunity in the host. An epitope, the smallest structural and functional unit of an antigen, binds specifically to an antibody or antigen receptor, to serve as key sites for the activation of adaptive immunity. The complexity and diverse range of epitopes are essential to study and map for the diagnosis of disease, the design of vaccines and for immunotherapy. Mapping the location of these specific epitopes has become a hot topic in immunology and immune therapy. Recently, epitope mapping techniques have evolved to become multiplexed, with the advent of high-throughput sequencing and techniques such as bacteriophage-display libraries and deep mutational scanning. Here, we briefly introduce the principles, advantages, and disadvantages of the latest epitope mapping techniques with examples for viral antigen discovery.

## Introduction

Adaptive immunity plays a vital role in the elimination of pathogens and the protection of organisms from re-infection. Adaptive immunity relies primarily on two types of lymphocytes, B cells and T cells, which mature in the bone marrow and thymus respectively and enter the peripheral lymphoid organs via the circulatory system (1). When foreign proteins (e.g. pathogens) are degraded, they are broken down into peptides and presented on the surface of the antigen presenting cell (APC) (2). When these peptides are detected via specific receptors on lymphocytes, the naive lymphocytes are activated, proliferate and differentiate into effector cells and memory cells (3, 4). The effector cells can specifically recognize ‘non-self’ foreign substances and either directly or indirectly eliminate pathogens or pathogen-infected cells. Different effector cells have their own specific functions. Plasma cells, the predominant effector B cells, secrete antibodies that specifically bind and recognize, thus mediating humoral immunity (5). Depending on their function, effector T cells are classified as cytotoxic T cells, helper T cells and regulatory T cells. Cytotoxic T cells bind and kill infected cells; helper T cells activate and maintain the function of other immune cells by producing a large number of cytokines; and regulatory T cells negatively regulate immune-mediated hyperinflammation (2). Memory cells also have the ability to specifically recognize ‘non-self’ foreign peptides, but unlike effector cells which die rapidly after the infection has been eliminated, memory cells can survive for long periods of time, up to 100 years (6). When re-infected with the same or a highly similar pathogen, memory cells are rapidly activated by the antigen and differentiate into effector cells, triggering a series of immune responses to clear the infection. This is the basis of immunological memory and is the main principle of vaccine immunity (7).

These foreign poly-peptides that stimulate adaptive immunity are collectively referred to as antigens. There are many different pathogens in nature, which in turn contain a variety of antigens. An effective antigen should have two main functions: immunogenicity, the ability of the antigen to efficiently activate the proliferation and differentiation of naive lymphocytes; antigenicity, the ability of the antigen to bind with high specificity to the immune effector cell (8). As antigen molecules are difficult to recognize by immune cells, epitopes are recognized by immune cells as an immune active region on the antigen. Epitopes, also known as antigenic determinants, are the smallest structural and functional units of an antigen molecule that bind specifically to an antibody or antigen receptor (8). Commonly, epitopes consist of 1-6 monosaccharides or 5-8 amino acid residues (B cells) or 8-11 amino acids (for T cells) (9, 10). Thus, an antigenic molecule contains more than one immunologically active region. Even the same pathogen can contain hundreds of different antigens.

B cells and T cells recognize epitopes via different mechanisms. B cells bind directly and specifically to antigens via the membrane surface immunoglobulins, called B cell receptors (BCRs), and differentiate into plasma cells and memory B cells with the same antigenic specificity. Plasma cells secrete antibodies that bind specifically to antigens and are important effector molecules in mediating humoral immunity against pathogens. The structure of an antibody is closely linked to its function. Antibody structures can be divided into variable region (V region) and constant region (C region) (11). The region of an antibody that has a highly variable amino acid composition is the V region. Both recombination and somatic hypermutation in the genes encoding immunoglobulins result in the high diversity of the V region, giving rise to diverse antibody repertoires (12). Theoretically, the human immune system can produce up to 10^26^ antibodies in different sequence combinations (13, 14). When a person is infected, a large number of antigenic epitopes of the pathogen continuously stimulate specifically recognized T/B cells. During massive proliferation and replication, activated B cells undergo somatic hypermutation, which alters the affinity of the antibodies. This occurs via gradual mutational optimization of complementarity-determining regions (CDR)-antigen interactions. During infections such as SARS-CoV-2, this has been shown to increase overtime to gradually evolve our antibody responses against a pathogen (15). Plasma cells that produce high-affinity antibodies are retained and proliferate, while plasma cells with poor-affinity are eliminated (16, 17). Antibodies can control and clear infections by directly neutralizing pathogens or toxins, activating complement, mediating antibody-dependent cellular cytotoxicity and antibody-dependent cellular phagocytosis (18). At high viral loads, viral genomes are highly susceptible to mutation, and these ongoing mutations may evade adaptive immunity and reduce its effectiveness (19).

The antigen recognition receptors on the surface of T cells are also membrane surface immunoglobulins and are known as T cell receptors (TCRs). Similar to BCRs, TCRs are also divided into V regions and C regions, and therefore TCRs have the ability to recognize, bind to and deliver activation signals to the cell (20). TCRs can generally only recognize antigens loaded into MHC molecules and so cannot recognize and bind to antigens directly. The key molecule, major histocompatibility complex (MHC), is therefore essential for processing and presentation of antigens by APCs. MHC-I primarily mediates antigen presentation of endogenous antigens and is expressed by all nucleated cells; MHC-II is expressed only by professional APCs (21). The antigens are degraded into short antigenic peptide fragments in APCs, usually 8-11 amino acids (22, 23). These subsequently bind to MHC and present themselves on the cell surface as an antigenic peptide-major histocompatibility complex (p-MHC), ultimately activating the T cells via TCR engagement (20).

Both B cells and T cells have the capacity to recognize various epitopes that suit a variety of functions. Epitopes may be further classified into the following classifications: linear epitopes and conformational epitopes, based on their structural distinctions. Linear epitopes, also known as continuous epitopes, consist of amino acid residues arranged in a continuous sequence. Conformational epitopes, also known as a discontinuous epitope, consists of amino acid residues that are not continuously arranged but are close to each other in spatial structure (24). While B cell epitopes can be linear or conformational, T cell epitopes are usually linear due to digestion and processing by APCs, though recently evidence for the importance of structural conformations in T cell epitope affinity has been observed (25).

During infection, diverse viral epitopes stimulate the host to produce a wide variety of antibodies and cytotoxic T cells (26, 27). Antigenic variation occurs between different strains and genotypes of the same virus. For example, Influenza A virus, whose major antigenic proteins, hemagglutinin and neuraminidase, vary between strains, generates subtype-specific immune responses (28). As viruses continue to replicate, mutations in the viral genome accumulate. Under immune selection pressure, viral mutants may acquire immune-evasion to survive, further increasing epitope diversity (29, 30). In addition, the epitopes targeted by antibodies and cytotoxic T cells vary between individuals, depending on a number of factors, such as age, history of previous infections, affinity maturation and the diversity of MHC genes (31–33). Adaptive immunity may not have the capacity to protect the host when Original Antigenic Sin, or failure to mount a response against an evolving pathogen, is present. As such, an optimal epitope is one that induces effective cellular and humoral immunity, no longer requires further adaptations, and plays a key role in the development of efficient vaccines or immunotherapies (8).

Epitope mapping technology is essential to clarify the natural antigenic landscape and evaluate the most potent and effective epitopes for an appropriate immune response. Common B cell epitope mapping techniques include Deep Mutational Scanning, peptide/protein microarrays, bacteriophage peptide/protein display and other peptide display techniques. Common T cell epitope mapping techniques are divided into three categories, peptide-MHC multimer-based, cell-based and yeast-based epitope mapping techniques. Here, we cover the latest in multiplexed epitope mapping technology (**Table 1**) and discuss their importance to the advancement of immunological therapeutics.

**Table 1:** Epitope mapping technologies.

Technique	Linear epitopes	Conformational epitopes	High resolution	High throughput	Time	Cost
B cell epitope						
Deep mutational scanning	Y	Y	Y	Y	++	++
Peptide/protein microarray	Y	N	N	Y	+	+
Bacteriophage peptide/protein display	Y	N^	N	Y	+	+
Bacterial surface display	Y	N^	N	Y	++	+
Yeast surface display	Y	N^	N	Y	+	+
T cell epitope						
Based on peptide-MHC multimers	Y	N^	N	Y	++	++
Based on cellular expression	Y	N^	N	Y	+++	++
Based on yeast display	Y	N^	N	Y	+	+
Structural analysis						
X-ray crystallography	Y	Y	Y	N	+++	+++
Nuclear magnetic resonance	Y	Y	Y	N	+++	+++
Cryo-electron microscopy	Y	Y	Y	N	+++	+++
Mass spectrometry	Y	Y	Y	Y	++	++

The table summarizes the characteristics of each epitope mapping technique. “Y” is yes and “N” is no. ^Partial conformation/structure of linear epitopes is tolerated. "+" indicates the degree of time/cost; sequentially from +,++ to +++ as the most expensive/longest time.

## B cell epitope mapping techniques

Traditional immunology has relied on specific antibodies generated against a single, well-defined, conformational epitope or a short linear section of a protein (often <50 aa’s). While whole proteins are sometimes utilized to generate antibodies, manual mapping of the antibody epitope must then occur through a range of somewhat arduous techniques. These techniques may provide high specificity and detail but they lack the capacity for en-bulk high-throughput mapping.

### Deep mutational scanning

Deep Mutational Scanning (DMS) utilizes gene mutagenesis, coupled with other techniques such as high-throughput sequencing, to obtain a saturated library of mutations covering antigenic epitopes (34, 35). DMS can be combined with peptide/protein display libraries to form a genotype-phenotype linkage, thus exploring the specific function of each amino acid located in the antigenic epitope by coupling to functional screens (35). Although DMS does not directly obtain structural information, it identifies specific amino acid residues that are involved in binding of linear or conformational epitopes. It may also identify key amino acid residues altering smaller conformational structures that interfere with the ability to bind other regions. Davidson et al. developed high-throughput shotgun mutagenesis-epitope mapping by combining DMS with peptide display techniques, to successfully identify at least 150 dengue virus epitopes (36). Using DMS, Francino-Urdaniz et al. identified five SARS-CoV-2 RBD escape mutants, of which K417, D420, Y421, F486 and Q493 are key sites mediating escape mutations (37). Greaney et al. also applied DMS to map escape mutations of SARS-CoV-2 and they predicted the possible RBD escape mutations under immune stress (38). Similarly, it can be used to study binding affinity profiles to assess the ability of commercially developed antibodies or vaccines to target novel mutants and in the case of SARS-CoV-2, evaluate restriction in the presence of new mutations observed in variants of concern (VOCs) (39, 40). Identifying the evolutionary trajectory of immuno-evasion can help in design of antibody therapies or vaccine design for generating vaccines resistant to potential escape (38).

### Peptide/protein microarrays

In 1984, Geysen et al. first proposed a technique for the rapid synthesis of peptides on the surface of immobilized carriers, followed by screening the immobilized peptides for antigenic epitopes using antibodies. Subsequently, Frank and Fodor et al. proposed new peptide synthesis techniques, SPOT synthesis and photolithography synthesis, respectively (41, 42). These two peptide synthesis techniques, especially SPOT synthesis, are more efficient and affordable compared to the traditional techniques of attaching the synthesized target peptide to the carrier surface.

Peptide/protein microarrays can be used to characterize antibody responses. Gallerano et al. prepared a set of microarrays covering HIV-1 clade C proteins and peptides that can be used to diagnose HIV and detect changes in specific antibody responses as HIV infection and treatment progress (43). Valentine et al. monitored changes in serum antibodies over time after Bacille Calmette-Guerin vaccine (BCG) by random peptide microarrays (44). Peptide/protein microarrays have also excelled in exploring the epitopes of emerging viruses, contributing to the understanding of the immune response induced by the virus. Several months after the initial SARS-CoV-2 outbreak, researchers managed to design a peptide microarray containing 966 representative SARS-CoV-2 peptides, based on the reference sequences of 10 proteins encoded by SARS-CoV-2 (45). Reactive IgM & IgG sera of COVID-19 patients in the early stages of infection were screened via peptide microarrays to obtain a B cell epitope map of the SARS-CoV-2 proteome, confirming four B cell epitopes predicted by bioinformatics and identifying an epitope located within the binding region of the SARS-CoV-2 RBD to the ACE2 receptor that may stimulate B cell neutralizing antibody production. Li et al. constructed a peptide microarray covering the SARS-CoV-2 spike protein and tested a large range of sera from COVID-19 patients (46). They evaluated an asymptomatic population and a control population and identified several epitopes of high diagnostic value, providing new epitopes for a more accurate, efficient and cost-effective diagnosis of SARS-CoV-2. Schwarz et al. applied a high-density peptide microarray including the whole SARS-CoV-2 proteome for a longitudinal study of serological changes in patients (47). Their results suggested that circulating serum IgA has a very short lifespan compared to IgG, with a late peak for induction and rapid decay, whereas circulating IgG can be present in serum at high titers for a long period of time. Using peptide microarrays covering SARS-CoV-2 spike and nucleocapsid, Voss et al. found that high levels of antibodies against S-811-825, S-881-895 were strongly associated with poor disease outcomes whereas antibody levels against N-156-170 were negatively associated with poor outcomes (48).

The Sengenics KREX chip is a commercially available protein microarray that immobilizes proteins with full-length sequences, correctly folded carrier and functional validation. Due to the presence of biotin carboxyl carrier protein (BCCP), BCCP-tagged biotinylated KREX proteins can only be immobilized on the chip if the carrier protein is correctly folded (49). The majority of mis-folded proteins are believed to upset the BCCP tag and therefore reduce the loading of misfolded proteins. Using the Sengenics KREX microarray, Smith et al. not only found a strong correlation between antibody titer and disease severity and age, but also identified an immunodominant epitope for SARS-CoV-2 located in the C-terminal domain of nucleocapsid protein (50).

The identification of important immunogenic epitopes of pathogens plays a key role in the development of targeted antibody therapies. Gu et al. used peptide arrays to screen the epitopes recognized by antibodies in the sera of patients at different stages of hepatitis B infection (51). They found that antibodies in the serum of hepatitis phase patients with alanine aminotransferase (ALT) flares recognized more epitopes than those in chronic infection phase, and that the number of epitopes recognized by antibodies in the serum rose as patients moved from chronic infection phase to hepatitis phase patients with ALT flares. Accordingly, they identified several epitopes that may be highly relevant to the treatment of chronic hepatitis virus infection as vaccine candidates.

Due to their flexibility, speed, relative affordability and high throughput, peptide/protein microarrays are widely used in the development of new diagnostics, therapeutic antibodies, and vaccines against pathogens. However, there are still many shortcomings with the automated use of peptide microarrays. Current techniques for constructing novel peptide/protein microarrays are costly and inefficient (52–55). When the artificial surface chemistry has inconsistencies, it may result in an uneven density distribution of peptides (56). Regional changes in intensity, therefore, do not necessarily imply changes in peptide/protein binding capacity but may result from discrepancies in surface chemistry, as such, additional controls and corrections to the data are required. Non-target proteins can also rapidly adsorb to the artificial surface, increasing background (57, 58). Non-specific proteins can increase false positive results yet also prevent the binding of the target protein to the immobilized peptide, leading to false negatives. To ensure that the peptide-antibody complex remains stable throughout, the times for washing, relative on-off rates etc., must be carefully validated to accurately quantify antibody binding, whereby data for low affinity interactions may be lost (59). Therefore, the relative advantages and disadvantages of peptide microarrays must be carefully evaluated depending on the experimental requirements.

### Bacteriophage peptide/protein display technology

To further meet the demand for mass production of peptides and multiplexing, peptide display technologies have emerged as a potential solution. Bacteriophage peptide display technology automatically and efficiently translates a large range of target sequences into peptides and presents them to the external surface with minimal extrinsic processing and as such is ideal for epitope mapping. Most phage display libraries utilize insertion of a small gene fragment as a fusion with a natural coat protein variant, thereby facilitating transcription, translation, and display on the surface. While large full-length proteins up to 50-80kDa can be expressed on the phage-surface, the insertion of small linear peptides allows for minimal bias between different inserted proteins and facilitates rapid and efficient processing when dealing with very large libraries (60). While not used for epitope identification, antibody identification often utilizes high diversity Fab fragment (~25kDa) libraries displayed on phage surfaces (61). In 1990, Scott and Smith developed the idea of using phage display technology to screen for epitopes (62). Later, Smith demonstrated that epitopes displayed on the surface of phages could be recognized and bound by specific antibodies (63). Phage display libraries have been made up to 10^11^ pfu/ml in size and can contain thousands of different peptides/proteins (64). Indeed, bacteriophages have been used to display functional SARS-Cov-2 Spike RBDs on the surface, coupled to functional screening, indicating a role for structural conformation of whole protein domains present on the phage surface (65). Additionally, phage-based protein scaffold libraries have already been developed for surface presentation of small proteins (66). This provides hope for utilization of phage-display technologies to display entire protein libraries suitable for epitope mapping.

With the development of sequencing technology in recent years, Larman et al. were the first to combine phage display, high-throughput DNA oligo synthesis and high-throughput sequencing to invent a more efficient and cost-effective technique called phage immunoprecipitation sequencing (PhIP-seq) for identifying antigenic epitopes recognized by antibodies in autoimmune diseases (67). Unlike traditional methods, the PhIP-Seq technique introduced defined amino acid motifs of a specific length representing a specific list of known pathogens. PhIP-seq does not require multiple rounds of biopanning for enrichment of antigenic epitopes; epitopes are enriched by PCR using barcoded PCR primers after phage-antibody immunocomplex precipitation (67, 68). Traditionally, the M13 phage is used extensively for phage protein display. Recently, the T7 phage is also becoming more accepted due to a high efficiency in expressing peptides and its ability to lyse the host cell, post-replication, thereby avoiding the effect of the host cell’s proteins on subsequent amplification (69–71). For peptide display, the PhIP-seq method has higher yields, lower costs, longer display peptides and higher quality compared to traditional peptide microarrays (68). Due to the high throughput of PhIP-seq and its ability for massive-multiplexing, it can be utilized to screen large numbers of epitopes in a simple liquid format compatible with robotics. PhIP-seq and other phage-display technologies, therefore, have a wide potential for applications in the field of virology, such as epidemiological surveys, risk factor-related surveillance studies, pathogenic diagnosis, and vaccinology.

Phage-display libraries may be used to quickly screen multiple different peptide or protein display libraries. One particular PhIP-seq library, Virscan, encodes a peptide display library covering the antigenic epitopes of the currently known human viral proteome (72). Due to this coverage of over one thousand viruses, Virscan profiled each individual’s infection history, both in terms of ongoing infections and past infection history. PhIP-seq was used to identify etiological evidence for infectious diseases of unknown etiology and can be combined with traditional serological testing techniques to improve diagnostic accuracy for targeted treatment (73–75). For example, Schubert et al. applied PhIP-seq and found that the cerebrospinal fluid of patients with acute flaccid myelitis (AFM) was enriched with antibodies against enterovirus epitopes, providing new evidence that enteroviruses are a potential cause of AFM (73). PhIP-seq can also screen for risk factors for infection that may be associated with disease or pathological states (31, 75–77). For existing vaccines, PhIP-seq provides long-term monitoring of vaccine efficacy, dosing, or comparison with naturally infected individuals to identify specific epitopes of antibodies produced by vaccination and thus guide vaccine improvement. For vaccines in development, PhIP-seq provides access to specific antigenic epitopes of induced antibodies (especially neutralizing antibodies) as well as epitopes that cross-react between either viruses or highly conserved epitopes, which may be important for the design of efficient and broad-spectrum vaccines (31). In addition, PhIP-seq can be combined with other techniques, such as alanine mutagenesis or mutation scanning, to improve resolution and help predict the likely mutational direction of the virus (31). Combining epitope information from PhIP-seq with the clinical history of the patient’s disease was used to help define and predict the severity of the patient’s condition (78).

Two limitations of the high-throughput phage display technique are the inability to display peptides or proteins that are post-translationally modified, such as glycosylation, SUMOylation etc., and when using synthetic peptide libraries or small protein libraries, the inability to display conformational epitopes associated with large proteins and complexes (over 50-80 kDa) (72). While larger domains and proteins may be expressed on bacteriophage surfaces, this does require optimization and validation in a manner not very consistent with unbiased, high-throughput workflows. This does provide leeway for bacteriophage-screening of conformational epitopes in the future, however, with optimization and development of new techniques. Additionally, the design of each new (large) phage display library requires considerable cost, particularly if using unique gene sequences, though this does depend on the library diversity. Randomized oligos and error-prone PCR may provide cheaper alternatives and synthetic library generation costs are quickly decreasing as gene synthesis techniques advance. Yet, with its’ ability to process large sample sizes, express diverse epitopes without the need to clone from source materials and the simultaneous mapping of a large number of functional epitopes (theoretically up to 10^11^), phage display techniques utilizing synthetically produced libraries still have an irreplaceable advantage (31, 64, 72).

### Other peptide/protein display techniques

Bacterial surface display is a common technique for displaying epitopes on the surface of bacteria. First, a suitable host is selected as the display platform (79). Gram-negative bacteria are often used as host cells because of their familiar genetic background. The bilayer structure of Gram-negative bacterium sometimes prevents the display of larger peptides on the surface of the cell membrane, whereas Gram-positive bacteria have only a single membrane and can facilitate the display of fusion peptides (80). Carrier proteins are then required to translocate the peptides to the bacterial surface. As such, eventual surface presentation may vary depending on the choice of carrier protein. However, both the choice of host cell and carrier protein requires careful consideration of the properties of the target peptide (79). The size of the peptide or protein to be displayed is of importance also, as very large proteins have more potential variation between proteins in the same library. Bacterial surface display is highly sensitive and specific for the diagnosis of pathogens, even compared to traditional serological tests with inherent signal amplification (81). It has been used to predict disease progression based on the variability in the specific epitopes of antibodies in patients with different levels of disease severity and efficiently detect pathogenic immune evasion caused by mutations thus can facilitate improved vaccine design (82).

Yeast surface display is similar to that of bacterial surface presentation. Coupling the epitope sequence to an anchoring protein sequence on the surface of the yeast host allows the epitope to be presented on the yeast surface. Yeast, unlike bacteria, is a eukaryotic cell and is therefore closer to mammalian cells in terms of protein folding, post-translational modifications and secretion mechanisms (83). For example, yeast expression of coronavirus RBD is highly similar to that expressed by human cells (84, 85). This gives yeast surface display an advantage for the expression and display of antigenic epitopes. In addition, yeast surface display can significantly improve enrichment and screening efficiency through Magnetic Cell Sorting (MACS) and fluorescence-activated cell sorting (FACS) (86). Yeast surface display in tandem with deep mutational scanning, and high-throughput sequencing, form a platform to provide efficient, accurate epitope mapping (87). Yeast display with DMS was previously used to map epitopes for Influenza A Virus Hemagglutinin (88). The advent of DMS and high-throughput sequencing together with yeast display suggests not only identification of antigenic epitopes of pathogens, but also prediction of potential mutation, thereby improving target site choice for vaccine and antibody therapies (38, 89–91). Limitations include the lower efficiency of transformation into yeast and that the diversity of peptides expressed by the yeast display system is approximately 10^8^-10^9^, significantly less than other peptide display techniques (92). Unlike bacteriophage display libraries that express from a single gene insertion site, multiple copies of a protein or peptide may be transformed and present on the same yeast surface at the same time, resulting in unwanted multivalent binding and affecting readouts for binding affinity (93).

In addition to phage display techniques, several other peptide display techniques have been developed and used for epitope mapping. A shotgun mutagenesis strategy was used to express SARS-CoV-2 Spike RBD mutants in mammalian cells for antibody mapping via 384-well plate high-content immunofluorescence (94). Due to the endogenous eukaryotic secretion mechanism, the structure and function of proteins/peptides displayed in mammalian cells are more similar to natural human proteins than via bacterial or yeast display. Mammalian cell display can also be used for high-throughput rapid screening of target proteins/peptides (95, 96). Ehling et al. performed single-cell sequencing of COVID-19 patient plasma cells to obtain antibody Ig genes and used them to construct a mammalian display library (96). The library was screened for SARS-CoV-2 binding by flow cytometry and deep sequencing in a high-throughput manner. Importantly, 43 antibodies were consequently identified to be specific for the SARS-CoV-2 antigen. Due to the limitations of low transfection efficiency, however, mammalian display libraries have relatively small library sizes (usually up to 10^7^). This limitation has been partially ameliorated with integration of CRISPR-Cas9, or another approach that utilizes libraries from immunized animals with initial antibody screening and maturation *in vivo* (97, 98).

## T cell epitope mapping techniques

Unlike B cell epitopes, T cell epitopes require the formation of an MHC-peptide (p-MHC) complex by an APC in order to be specifically recognized by the TCR. Overlapping peptide pools have previously been used for systematic screening and epitope identification of Infectious Bronchitis Virus in humans by direct stimulation in PBMCs, using readouts such as ELISpot or cytokine production measured by flow cytometry (99, 100). This method has the advantage of requiring functional surface recognition to trigger T Cell stimulation, with modified cell lines allowing identification of specific HLA-restriction, though it may require multiple rounds of screening to identify individual peptides, and to date, is yet to accommodate very large peptide libraries (101). While this complex multi-protein/peptide structure may be hard to mimic artificially, several techniques are still available for high-throughput T cell epitope mapping.

### Epitope mapping techniques based on peptide-MHC multimers

As this complex heteromeric MHC protein is normally folded inside the cell prior to loading a peptide (with additional help from other proteins), this key p-MHC complex formation is essential for T cell epitope mapping. The generation of libraries for T cell binding therefore goes well beyond just the generation of antigens. In the absence of co-stimulation, p-MHC has a very weak affinity for TCR(1-100 μM), dissociating rapidly and making it difficult to detect the interaction between TCR/p-HMC (102). Alman et al. pioneered the development of an Avidin-biotin-based p-MHC tetramer, which facilitated a stabilized interaction between the TCR and p-MHC (103). Subsequently, dimeric, pentameric and higher oligomeric forms of p-MHC multimers were developed. The classical method of p-MHC multimer staining involves coupling p-MHC multimers with fluorescent markers and incubating them with T cells, thereby identifying T cells that recognize the p-MHC multimers by traditional flow cytometry (103). To further improve the ability to screen T cells for epitopes, a technique combining many p-MHC multimers labelled with heavy metal ions instead of fluorescence, and then screening by mass spectrometric analysis (cytometry by time-of-flight, or CyTOF) was developed (104). Multiple permutations between different isotopes or between isotopes and specific antibodies have been investigated to increase the number of simultaneously detected peptides, though the technique is currently limited to 96 barcodes (105–107). Neither fluorescent nor metal tags are able to cover the depth of T cell epitope diversity, which is greater by several orders of magnitude, therefore failing to perform true high-throughput epitope mapping. In an attempt to achieve high throughput screening of T cell epitopes, unique DNA barcodes have been used instead of fluorescent or metal tags (108). Combinations of different bases to generate unique DNA barcodes, detected via NGS sequencing, generate a high enough degree of diversity, allowing each sequence of p-MHC to contain a unique label. The DNA barcode-p-MHCs are then fluorescently labelled to screen for functional binding to T cells and consequently determine the epitope sequence. Saini et al. used p-MHC multimers with DNA barcodes to specifically detect T cell epitopes that map to SARS-CoV-2, ultimately identifying 122 epitopes targeted by CD8+ T cells (26). The vast majority of these epitopes were derived from open reading frames. This is an important guide for vaccine design to induce specific T cell immunity.

### Epitope mapping techniques based on cellular expression

When cells come in contact, the phenomenon of sharing and transferring membranes and membrane-associated proteins between cells is called trogocytosis (109). When T cells bind to APCs, the T cells acquire the membrane and associated proteins from the APC (one-directional) (109, 110). However, genetically engineered APCs can convert the unidirectional trogocytosis of T cells into bidirectional trogocytosis (111). An epitope mapping technique was devised by Joglekar et al. using this same principle (112). T cells expressing TCR specifically recognize and label p-MHC on the APCs displaying cognate peptide. Currently, the method has a labeling rate of 70% for identifiable APCs, while unrecognized APCs are not labeled. The labeled APCs (with successful binding) are then screened by FACS and sequenced to obtain specific epitope information. Unlike other techniques that are limited by the development of MHC alleles, this method is based on direct, natural, interaction between cells. In theory, it can easily be applied to a wider range of TCRs or MHC alleles without optimization. This also implies that the technique allows for high throughput screening and detection of a more diverse range of epitopes.

In addition, they have developed another epitope mapping technique that uses a chimeric receptor called the signal transducer and antigen presenting antigen bifunctional receptor (SABR) to identify TCR-p-MHC interactions (113). SABR, a p-MHC multimer (extracellular) attached to a signal transducer on the intracellular side. The target cell requires a readout such as GFP. When the TCR-p-MHC interaction occurs, SABR transmits the signal into the cell to stimulate the transcription and translation of GFP. Ultimately, the target cells are screened for GFP expression by flow cytometry and further epitope sequencing from APC genomic DNA is performed. SABR also allows high throughput screening, currently up to 10^6^ epitopes, but significantly less than the diversity of the yeast display library. False positive results have occurred in some cases and further refinement of the technique is required to improve the specificity of the assay.

Using both approaches, homologous epitopes of novel TCRs isolated from melanoma patients were successfully retrieved. The feasibility of these two cell-based epitope mapping techniques in exploring homologous epitopes of TCRs was demonstrated, to guide the site of action of immunotherapy (112, 113). The technique exhibited a good potential for high-throughput viral epitope mapping by p-MHC deep sequencing. Liquid chromatography-tandem mass spectrometry (LC-MS/MS)-based epitope mapping techniques have been used to identify naturally expressed HIV peptides on the surface of APCs, combined with identification of HLA-restricted T cell recognition and consequent identification of p-MHC’s associated with favorable clinical outcomes (114–116). However, these methods fail to identify all identify all peptides expressed on the surface of cells and the diversity of peptides identified appears relatively small.

T-scan is a genome-wide approach that allows comprehensive scanning and discovery of cytotoxic T cell epitopes (117). The key point of this technology is that activated cytotoxic T cells secrete a serine protease, granzyme B (GzB), which specifically targets APCs and cleaves proteins to promote apoptosis (118). Based on the specific induction of GzB, they developed a GzB reporter system. When cytotoxic T cells are specifically activated by p-MHC, GzB is released into the cell, triggering cleavage of a GzB-linker and activating fluorescence. Thus, activated target cells can be further isolated by FACS and specific epitopes can be identified from the APCs using PCR against the p-MHC coupled with NGS. Using T-scan, they identified four undiscovered antigenic regions and mapped the TCR-epitope binding interface (117). T-scan has now been applied to the study of T cell epitopes of SARS-CoV-2. Unbiased screening of cytotoxic T cell epitopes from COVID-19 patients has identified T cell epitopes that are highly conserved among coronaviruses and mutant epitopes of SARS-CoV-2 leading to decreased cellular immunity (119). These studies provide ample evidence that the T-scan can help guide the design of viral vaccines at key epitopes.

### Epitope mapping technique based on yeast display

As described earlier for B cell epitopes, the structure of proteins displayed through yeast is highly similar to that of mammals, and therefore yeast display can be applied for high-throughput epitope mapping of T cells. Yeast libraries are specifically generated with a high diversity of peptides though unlike B cell epitope mapping, the display libraries utilize a fusion protein with a cell wall anchoring protein expressed on the cell surface. This gives each yeast the ability to display a unique p-MHC on its surface (120). The yeast display library is then incubated with fluorescently labelled soluble TCRs or TCRs combined with magnetic beads and sorted by multiple rounds of FACS or magnetic aspiration to identify p-MHC-TCR complexes and sequencing of the peptide-encoding gene present in the yeast (121–123). After obtaining information on potential T cell epitopes, in-depth data analysis is performed to match functional hits from the library with observed natural T cell epitopes. Currently, human MHC molecules that have been displayed by yeast include HLA-DR15, HLA-DR1, HLA-DR4, HLA-DQ6 and HLA-A*02:01 etc (120–123).

Chiou et al. used a yeast peptide-HLA A∗02:01 display library to identify the non-small cell lung cancer (NSCLC) epitope, TMEM161A, overexpressed in cross-reactive epitopes of Epstein-Barr virus and *Escherichia coli*, suggesting that the presence of antigenic cross-reactivity between tumor and virus may be a novel feature in oncogenesis (124).

In contrast, the yeast agglutination-mediated TCR antigen discovery system (YAMTAD) enables rapid and efficient confirmation of p-MHC-TCR interactions even in the absence of TCR tetramers with the aid of yeast display and engineered yeast mating (125). The basic principle is that the TCR, as well as the MHC are displayed on the surfaces of mating-matched MATa/MATalpha brewer’s yeast cells. If the TCR displayed on a MATa yeast recognizes the homologous p-MHC displayed on a MATalpha yeast, mating is induced. By carrying a mating-specific fluorescent reporter and complementary amino acid-assisted nutritional markers, it allows mating yeast to be identified by either FACS or diploid selection.

Other techniques for T cell epitope identification have also been developed recently, including lentiviral expression libraries containing antigens for binding of TCRs and BCRs, coupled to scRNA-Seq for simultaneous identification of both antigen (post-integration into the genome) and the TCR/BCR itself from the same cell (126). This technique offers high throughput screening, though how many antigens fail to be expressed/bind TCR/integrate etc., due to technical reasons is still unknown. Some of the current challenges in epitope mapping of B and T cells include the difficulty in obtaining purified antigens and p-MHCs that reflect their natural state, the continued lack of exploration of epitopes targeted by MHC-II-restricted TCRs, the partial limitation of sensitivity and specificity of epitope mapping, and uneven distribution of single-point and combined-DMS hits.

## Low throughput structural analysis

Predictive structural modelling of proteins with the likes of AlphaFold has dramatically improved accessibility of computationally predicted structures. However, epitopes and conformational epitopes, are often flexible regions that do poorly with structural prediction due to the lack of constraints. Additionally, modelling and docking of peptides inside the MHC-groove and between MHC, peptide and TCR are still relatively poor predictors of true binding dynamics. Recent advances in machine-learning driven TCR-epitope binding are improving, however, structure-based epitope mapping techniques are still an indispensable tool for understanding many epitopes (127). While these technologies may generate high-resolution, fine-detail mapping, they all suffer from workflow limitations, namely that they are largely low-throughput. While the advent of liquid handling robotics, high-throughput chemistry and enhanced frameworks for automated antibody crystallization are improving this field, the acquisition time and cost of data collection remains the biggest limitation (128).

X-ray crystallography not only enables the construction of 3D structural models of antigen-antibody complexes, but also the detailed description of epitope, paratope and the interaction forces between them. Koenig et al. designed four SARS-CoV-2 neutralizing nanobodies and identified two different epitopes by x-ray crystallography, among which one bound by VHH E overlaps highly with ACE2 (29). However, the construction of three-dimensional (3D) crystal structures by X-ray crystallography is not easy. Stable, high-quality crystals, complex data processing, specialized technicians, and expensive equipment greatly limit the application of x-ray crystallography for high-throughput epitope mapping (129–131). Cryogenic electron microscopy (Cryo-EM), which collects the scattered signal of an electron beam after it has passed through ice layers, uses less starting material, can image protein complexes in solution and can image antibody-epitope complexes, is used to re-construct detailed 3D structures (132). Recent advances in Cryo-EM combined with powerful computational advances are constantly increasing the maximum obtainable resolution (94, 133). 384-well plate-based immunofluorescence combined with negative stain EM also allowed semi-high throughput screening for epitope mapping. By determining the 3.1 Å structure of the neutralizing antibody 4A8 bound to SARS-CoV-2, Chi et al. proposed that the Spike NTD could serve as a new epitope for SARS-CoV-2 treatment (134). Like X-Ray crystallography, EM is costly due to expensive equipment, manual screening of samples, long data acquisition and computational analysis. While it may be limited to hundreds of proteins at a time, Cryo-EM does not require the formation of crystals from proteins and the amount required is much smaller than X-ray crystallography. Together, it now provides complementary epitope information together with X-ray crystallography, rather than being used as a stand-alone technique (135, 136).

Mass spectrometry analyzes charged molecules or ions by mass-to-charge ratio (m/z). Hydrogen/deuterium exchange, in the core binding region of antibody-bound epitope, is significantly lower than in the unbound region, allowing a readout for specific information on conformational epitopes (137). Mass spectrometry therefore has the ability to map both types of epitopes (138, 139). It has been used to map polyclonal antibody epitopes in the sera of rabbits receiving *Neisseria meningitidis* vaccine and thus guide the improvement of vaccines (140). Combined with other structural techniques it can identify details of the epitope-paratope interface in antibody-epitope complexes (141, 142). Mass spectrometry is more rapid, sensitive and somewhat high-throughput compared to other structural techniques (143, 144).

## Prospects for the future

**Figure 1:**
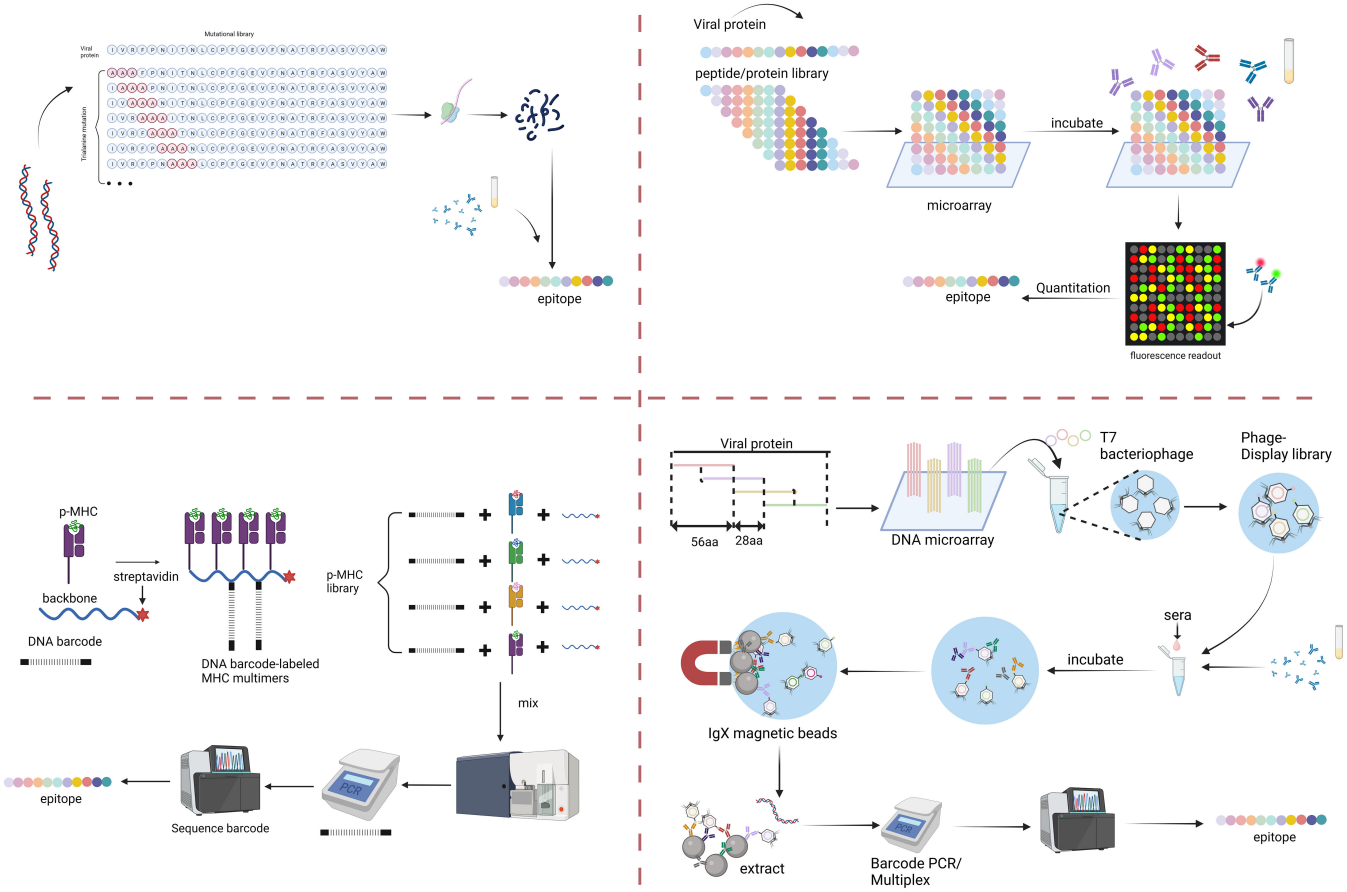
Multiplexed serology for viral epitope mapping. Top left: Alanine Scanning-Deep Mutational Scanning (DMS) of dsDNA to generate multiple epitope variants. Top right: peptide/protein arrays with hundreds/thousands of proteins spotted onto glass array slides and quantified by fluorescent antibody binding. Bottom right: bacteriophage display library, such as Virscan, generating single bacteriophage with single peptides, then quantified via NGS sequencing post-immunoprecipitation. Bottom left: p-MHC loaded multimers screened in a similar way to display libraries, coupled to NGS of attached DNA barcodes for epitope identification.

Adaptive immunity generates long-term immunological memory and can stimulate powerful pathogen clearance; as such, antigenic epitopes play an important role in immunotherapy and vaccinology as a ‘switch’ to stimulate initiation. Epitopes associated with B cells can generally be characterized by the specific binding to antibodies generated by B cells. T cell epitopes are generally considered to be more simple, smaller epitopes compared to antibody binding, but their recognition is tightly associated with MHC loading and presentation. Both are associated with high-specificity and a great diversity of potential epitopes. This in turn increases difficulty for identification of specific epitopes and leads to several differences in epitope mapping techniques between B and T cells. Despite this, many mapping tools have been developed, allowing high-throughput epitope analysis (~10^10^-10^14^) coupled with classical structure-based techniques for fine conformational identification. T cell epitopes require the design of peptide libraries based on specific MHC molecules which poses difficulties due to the high polymorphisms of MHC molecules and more complex binding dynamics. Still, many tools for T cell epitope mapping have been utilized, although on a much smaller scale than those for B cells, to obtain high-resolution epitope information. These techniques, summarized in **Figure 1**, have been used to map specific epitopes in viruses such as IAV, DENV, HepC, EBV, Enterovirus, HIV, SARS-CoV-1, SARS-CoV-2 and even tumor antigens. The combination of epitope mapping together with utilization of large TCR or BCR-databases, may also reveal further insight into immunological antigenic landscapes (145).

The combination of different technologies may also accelerate the fine-mapping of important antigenic regions. As Next Generation Sequencing (NGS) becomes cheaper and more high-throughput, it facilitates more comprehensive characterization of higher diversity libraries (72). DMS, in conjunction with other epitope mapping tools, identifies possible mutant epitopes susceptible to viral immune escape, thereby helping to avoid vaccines targeting epitopes located in potentially mutated regions (31, 37). DMS provides a platform for studying the rapid evolution of viral proteins, and can be used to investigate potential new variants, prior to them being observed in nature. The combination of DMS with scRNA-Seq also helped identify specific T cell clones against functionally relevant viral epitopes. Recent research into post-vaccination responses against SARS-CoV-2 have highlighted the need for thorough investigation of both B cell and T cell responses. Techniques combining simultaneous B- and T-cell mapping would then also be highly beneficial. Potential combinations of the highly diverse phage display libraries with other mapping readouts may also accelerate identification of relevant viral epitopes for vaccine design.

Current techniques for epitope mapping have evolved towards large scale epitope screening, combined with lower cost, higher sensitivity, and increased specificity. Advances in sequencing, scRNA-Seq, barcoding, surface display libraries, DMS and cell-free binding have all rapidly expanded our knowledge of epitopes. Further utilizing this high-throughput data with machine-learning algorithms may also facilitate optimal epitope prediction methods, with higher success rates than what is currently observed, to advance targeted vaccine design. This will also help to explore the general immune response process during viral disease, autoimmune disease, tumor progression and provide future guidance for disease diagnosis, vaccine development and immunotherapy (146–149). These massively-multiplexed epitope mapping platforms are now readily accessible, with decreasing costs and increased availability of reagents and techniques, such that they are now more applicable to a wide-range of scientific research needs.

## Author contributions

HD & AI wrote and composed the manuscript, sourced references and summarized appropriately. AI conceptualized the idea. Both authors contributed to the article and approved the submitted version.
